# Malposition of a pulmonary artery catheter in the left ventricle: a case report

**DOI:** 10.1186/s40981-023-00622-y

**Published:** 2023-05-24

**Authors:** Toshiyuki Nakanishi, Shohei Kato, Tetsuya Tamura, Eisuke Kako, Kazuya Sobue

**Affiliations:** Department of Anesthesiology and Intensive Care Medicine, Nagoya City University Graduate School of Medical Sciences, 1 Kawasumi, Mizuho-Cho, Mizuho-Ku, Nagoya, Japan

**Keywords:** Blood gas analyses, Complications, Pulmonary artery catheterizations, Transesophageal echocardiography

## Abstract

**Background:**

Placement of pulmonary artery catheters may be associated with a variety of complications. We present a case where a pulmonary artery catheter was accidentally advanced into the left ventricle by perforating the intraventricular septum.

**Case presentation:**

A 73-year-old woman underwent mitral valve dysfunction. A pulmonary artery catheter could not pass the tricuspid valve under general anesthesia, which was manually advanced via the right ventricle during surgery. After valve replacement, systolic pulmonary artery pressure was higher than radial arterial blood pressure. Transesophageal echocardiography (TEE) revealed the tip of the catheter in the left ventricle. The catheter was withdrawn and then advanced to the pulmonary artery under monitoring of TEE. Transseptal shunt flow gradually decreased and finally disappeared. The surgery was completed without additional procedures.

**Conclusions:**

Although ventricular septal perforation is rare, it should be recognized as a potential complication of pulmonary artery catheter insertion.

## Background

Pulmonary artery catheters (PACs) are used to continuously monitor cardiac output, mixed venous oxygen saturation, and multisite right heart pressure. Although several studies have shown no benefit of the use of a PAC on mortality and morbidity in patients undergoing cardiac surgery [1, 2], it has been used in selected patients and centers [3, 4].

There are several complications associated with the use of PACs, with pulmonary artery perforation being one of the most serious [5]. Puncture-related complications, such as pneumothorax, arterial injury, and arrhythmia, may also occur during PAC insertion. In addition to these well-known complications, some investigators have reported rare but life-threatening complications, including right ventricle (RV) perforation [6–8], in which the tip of the PAC may perforate the free wall of the RV. This RV perforation may be detected by cardiac tamponade or direct observation of the PAC protruding from the RV during surgery [6–8].

In this report, we describe a case with malposition of a PAC in the left ventricle (LV). Malposition of a PAC in the LV may not cause hemodynamic change and may not be directly observed. Using transesophageal echocardiography (TEE) images and pressure waveforms, we diagnosed the malposition of the PAC. We also discuss the management strategy for this rare complication.

## Case presentation

A 73-year-old woman (height 146 cm, body weight 32 kg) with a history of hyperlipidemia and asymptomatic cerebral infarction was referred to our hospital for congestive heart failure due to mitral regurgitation (MR) and tricuspid regurgitation. She underwent mitral valvuloplasty with artificial tendon cords and annuloplasty and tricuspid annuloplasty through a small right thoracotomy. MR recurrence due to a rupture of the artificial tendon cord was noted 2 days after surgery, and mitral valve replacement with a median sternotomy and extracorporeal circulation was scheduled.

The patient was preoperatively medicated with carvedilol, tolvaptan, azosemide, atorvastatin, and perindopril. Preoperative transthoracic echocardiography (TTE) showed severe MR with a ruptured artificial tendon of the posterior papillary muscle of the mitral valve. Blood tests revealed anemia (hemoglobin 8.8 g/dL), and 12-lead electrocardiogram indicated monofocal supraventricular paroxysmal contraction, LV hypertrophy, and left atrial overload.

After the induction of anesthesia with midazolam, fentanyl, remifentanil, and rocuronium, we intubated the patient’s trachea and inserted a TEE transducer. We placed a 9-Fr sheath via the right internal jugular vein under ultrasound guidance and attempted PAC insertion under TEE guidance. However, the PAC could not pass the tricuspid valve, possibly because of the narrow tricuspid area after the annuloplasty. We therefore decided to start surgery with the PAC tip remaining in the right atrium (RA) and asked the surgeon to reposition it intraoperatively into the RV.

**Fig. 1:**
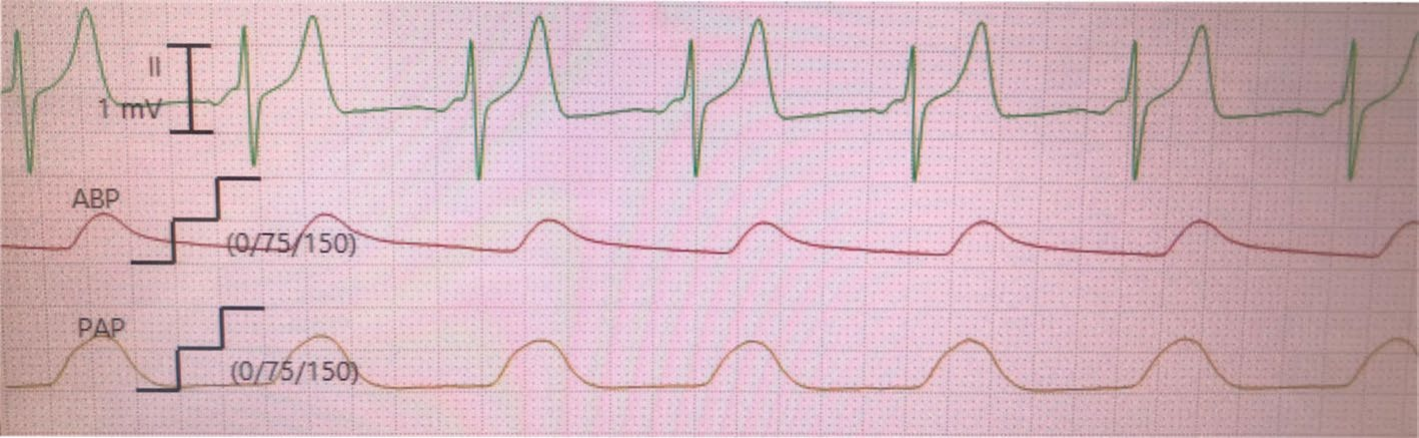
Pressure waveforms at the left radial artery and the tip of the pulmonary artery catheter. The systolic PAP (approximately 100 mmHg) was higher than that of ABP (approximately 80 mmHg). The diastolic PAP (approximately 10 mmHg) was lower than that of ABP (approximately 40 mmHg). ABP, arterial blood pressure; PAP, pulmonary artery pressure

**Fig. 2:**
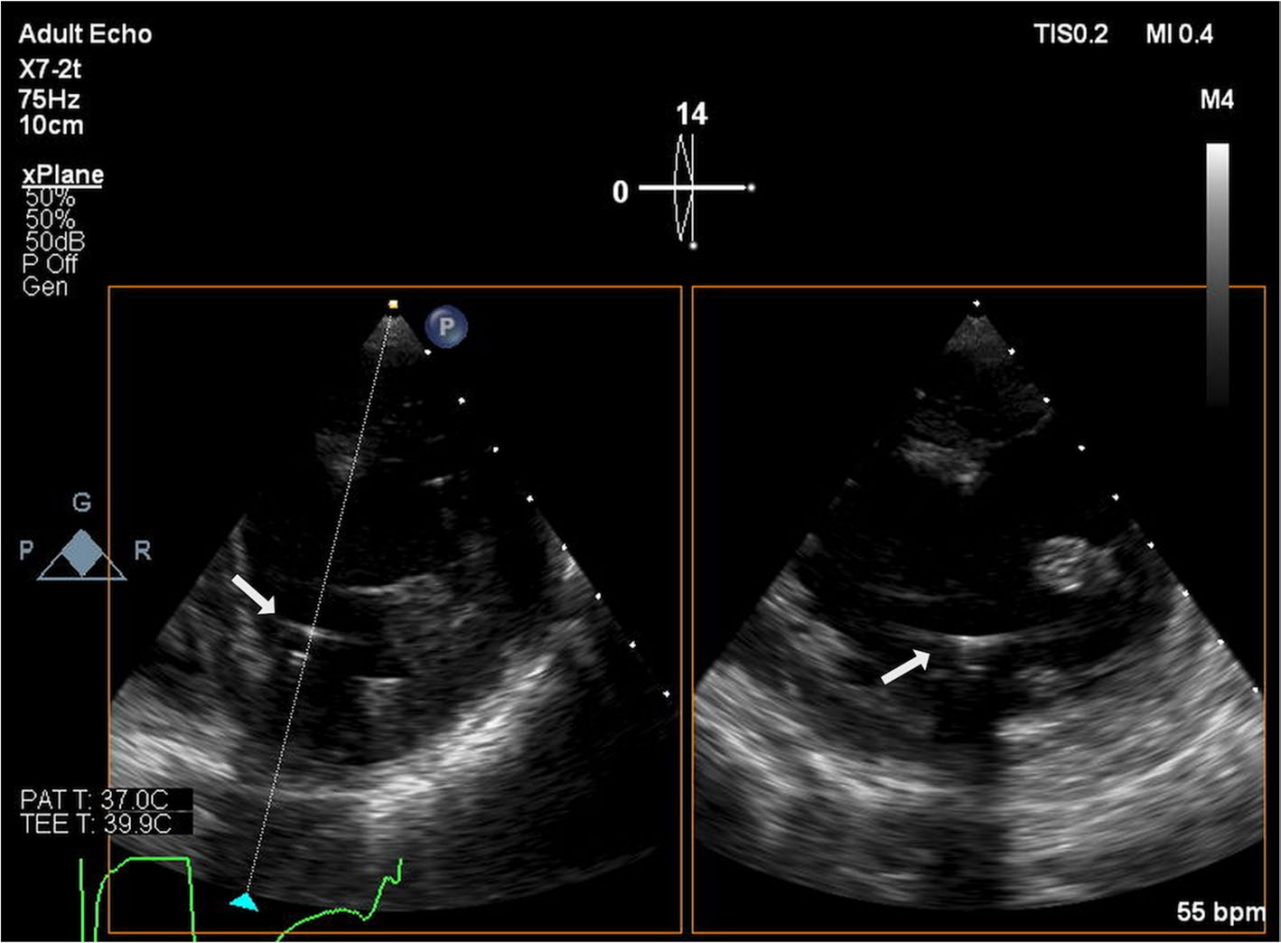
Transesophageal echocardiography images of pulmonary artery catheter malposition in the left ventricle. Biplane images were obtained using the transgastric approach. The left and right images are a short-axis view and a two-chamber view of the left ventricle, respectively. The pulmonary artery catheter balloon (white arrows) was inflated with air, allowing observation of the high-intensity reflection and the acoustic shadow behind it

After the mitral valve was replaced (with a 27-mm tricuspid bioprosthetic valve), the surgeon manually advanced the PAC from the RA into the RV. At this time, the balloon at the tip of the PAC remained deflated. After closure of the RA, we initiated pulmonary artery pressure (PAP) monitoring, which showed higher systolic blood pressure and lower diastolic blood pressure than arterial blood pressure (ABP) at the left radial artery (Fig. 1). The transgastric short-axis view of the TEE showed an inflated balloon at the tip of the PAC in the LV, indicating that the PAC had perforated the ventricular septum and migrated from the RV to the LV (Fig. 2).

**Fig. 3:**
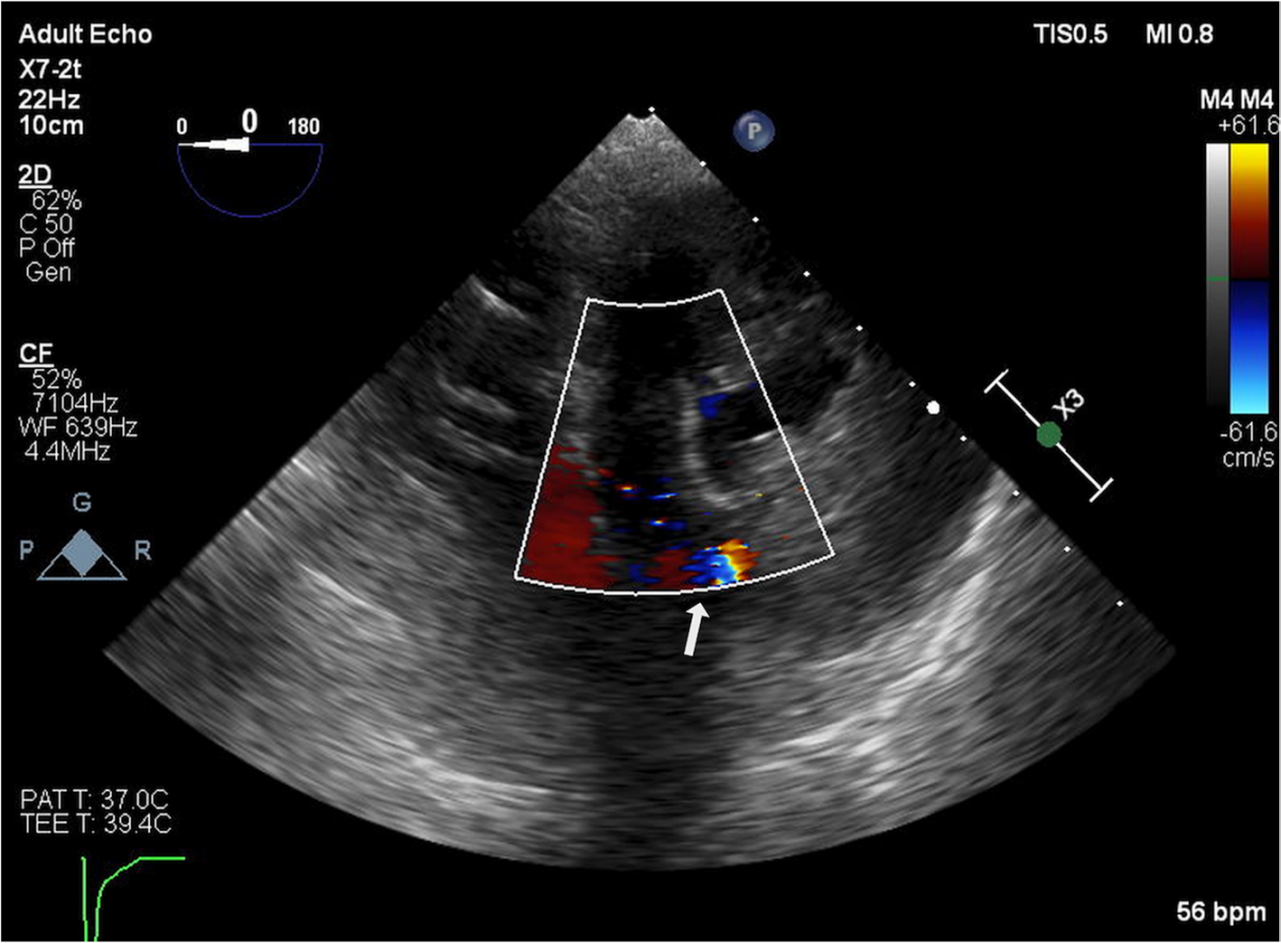
Colored flow Doppler image of the ventricular septal flow immediately after withdrawing the migrated catheter. The transgastric left ventricular short-axis view shows an accelerated blood flow (white arrow) in the 7- to 9-o’clock direction of the left ventricle wall

After discussion with the surgeon, the PAC balloon was first deflated, and the tip of the PAC was pulled out into the RV without much resistance. The PAC was then advanced to the right pulmonary artery under TEE guidance. At that time, the PAP value was 38/8 (18) mmHg [systolic/diastolic (mean)], indicating within the normal range. Using TEE, we evaluated the shunt blood flow through the ventricular septum. Initially, we observed a colored flow through the ventricular septum, but it gradually decreased and finally disappeared (Fig. 3). We performed blood gas analysis to confirm no oxygen step-up between the right and left hearts. The partial pressures of oxygen were 49.9 mmHg and 47.4 mmHg at the RA and pulmonary artery, respectively. We concluded that there was no significant residual ventricular septal shunt; therefore, cardiopulmonary bypass (CPB) was weaned, the patient’s chest was closed, and the operation was terminated. The duration of CPB and surgery was 169 min and 301 min, respectively.

The patient was admitted to the intensive care unit (ICU) under sedation and positive-pressure ventilation. After we confirmed there was no postoperative bleeding or circulatory problems, the patient’s trachea was extubated on the same night. On the first postoperative day, the PAC was removed after we reconfirmed that there was no oxygen step-up (37.3 mmHg and 35.2 mmHg at the RA and pulmonary artery, respectively). One week after surgery, postoperative TTE showed no residual shunt at the ventricular septum. The patient was discharged from the ICU on postoperative day 2 and transferred to another hospital for rehabilitation on postoperative day 13.

## Discussion

We experienced a case in which a PAC was advanced directly into the RV, perforated the ventricular septum, and migrated into the LV. Continuous TEE observation of the ventricular septum and assessment of oxygen step-up using simultaneous blood gas measurements in the RA and pulmonary artery allowed us to complete the procedure without the need for additional surgical intervention.

In our case, TEE revealed the malposition of the PAC in the LV and perforation of the ventricular septum. Because the ventricular septal wall is thicker than the free wall of the RV, the incidence of ventricular septal perforation may be extremely low. To our knowledge, there has been only one previous report of ventricular septal perforation by a PAC [9], where a PAC penetrating the LV-free wall was detected via direct vision after pericardiotomy. The perforated LV wall was then repaired with a suture. Contrary to the case reported by Benito-Saz et al., a PAC that remains in the LV, as in our case, cannot be directly visualized, making early detection difficult and resulting in subsequent free wall rupture of the LV. Because TEE provides the ability to visualize the tip of the PAC, it may play a critical role in the detection of PAC malpositioning.

Based on the absence of ventricular septal shunt flow on TEE and the step-up in the partial pressure of oxygen between the RA and the pulmonary artery, we determined that there was no significant residual shunt. In addition to the case report by Benito-Saz et al., there have been several reports of RV pacemaker leads perforating the ventricular septum and LV-free wall [10, 11]. In these previously reported cases, the LV perforation was surgically repaired, but the ventricular septum was not. In our case, we also did not perform any surgical intervention on the ventricular septum based on TEE observations and blood gas analysis. In cases of inadvertent ventricular septal perforation, TEE and partial pressure of oxygen monitoring can help confirm the absence of a residual shunt.

When the insertion of the PAC into the RV fails, as in our case, anesthesiologists may ask the surgeon to advance the PAC through the tricuspid valve during surgery. In our case, the balloon of the PAC advanced into the RV was deflated, which might have predisposed to ventricular septal perforation. When advancing the PAC into the pulmonary artery in cooperation with the surgeon, inflating the balloon and palpating the pulmonary artery may help avoid perforation of the ventricular septum. It is also definitely crucial to observe the PAC concurrently with TEE.

In summary, we report a case in which a PAC was advanced directly into the surgical field and perforated the ventricular septum from the RV to the LV. Although the incidence of ventricular septal perforation is rare, it should be considered a possible complication of PAC use.

## Data Availability

Not applicable.

## Abbreviations

ABP: Arterial blood pressure
CPB: Cardiopulmonary bypass
ICU: Intensive care unit
LV: Left ventricle
MR: Mitral regurgitation
PAC: Pulmonary artery catheter
PAP: Pulmonary artery pressure
RA: Right atrium
RV: Right ventricle
TEE: Transesophageal echocardiography
TTE: Transthoracic echocardiography
